# Fortification of Orange and Apple Juices with Ferulic Acid: Implications for Food Safety and Quality

**DOI:** 10.3390/foods13203288

**Published:** 2024-10-16

**Authors:** Maribel Abadias, Gloria Bobo, Marina Anguera, Jordi Ortiz-Solà, Ingrid Aguiló-Aguayo

**Affiliations:** Institute of Agrifood Research and Technology, IRTA, Postharvest, Edifici Fruitcentre, Parc Agrobiotech Lleida, Parc de Gardeny, 25003 Lleida, Catalonia, Spainmarina.anguera@irta.cat (M.A.); jordi.ortiz@irta.cat (J.O.-S.); ingrid.aguilo@irta.cat (I.A.-A.)

**Keywords:** *Listeria monocytogenes*, antioxidant, polyphenol content, minimally processed

## Abstract

In recent years, the awareness of healthier lifestyles among consumers has driven to an increased interest in more natural, nutritious, and low-processed foods. Ferulic acid, one of the most abundant phenolic acids in plants, has demonstrated a wide spectrum of antimicrobial activities and a range of biomedical effects, including antioxidant, antiallergic, hepatoprotective, anticarcinogenic, anti-inflammatory, and antithrombotic, among others. The objective of this work was to study the antilisterial effect of ferulic acid (FA, 1500 mg/L) on fresh, ready-to-eat orange (FOJ) and apple (FAJ) juices and evaluate its effect on product quality. The results showed that FA reduced the *Listeria monocytogenes* population after 9 days of storage at 4 °C, but no effect on the indigenous microbiota was observed. The titratable acidity and color significantly changed. The antioxidant capacity and total phenolic content significantly increased with the addition of FA, being at least two times greater in fortified juices. FAJ and FOJ containing FA were scored lower (6.8 and 5.7 on a 9-point hedonic scale, respectively) than their respective controls. Overall, our results demonstrated that FA treatment could be a useful strategy to maintain the safety of fresh apple and orange juices and increase the antioxidant activity and phenolic content. The potential industrial applications and health benefits of the fortification of fruit juices with FA should be further explored.

## 1. Introduction

In recent years, the awareness of healthier lifestyles among consumers has driven an increased interest in more natural, nutritious, and low-processed foods. Fruits and vegetables are sources of vitamins, antioxidants, minerals, and fiber and have low protein and fat content; thus, this tendency is accomplished. Moreover, experts have recommended a daily intake of 400 g of fruits and vegetables per day [1] because of their beneficial effect on health, but most of the population hardly ever consumes this amount. Fruit juices or smoothies with high vitamin, antioxidant, and mineral content and low sugar content could be an easy way to increase intake, and their demand has increased over the last decade. However, low-processed or untreated fruit beverages, even though they are usually acidic products, have a short shelf life, mainly due to spoilage associated with microbial growth [2], which can occur under refrigeration conditions. Unpasteurized apple and orange juice have been linked to several outbreaks worldwide, mainly due to *Salmonella* spp. and *E. coli* O157:H7 [3]. Although no outbreaks of listeriosis have been linked with the consumption of juices, *Listeria monocytogenes* is a pathogen of concern owing to its high rate of mortality and its ability to survive in acidic foods under refrigeration conditions. In addition, *L. monocytogenes* prevalence throughout the fresh produce supply chain has been found to range from 0 to 61.1% [4], indicating the potential risk of contamination of the raw material used to produce the juice with this pathogen.

Thermal treatments are commonly applied to ensure safety, inactivate enzymes, and prolong the shelf-life of juices. However, several studies have shown that high temperatures could cause chemical and physical changes that affect sensory properties and reduce the content or bioavailability of some nutrients [5]. Several nonthermal processes (e.g., high-pressure processing, ultrasound, radiation) have been investigated in recent decades to overcome these drawbacks [6]. Another alternative to thermal treatments is the use of phenolic acids, which could have several biological activities on juices, such as antimicrobial, antioxidant, or fortification effects. Beverages such as juices are convenient and easy to drink, palatable, and with many variations, making them good vehicles for the incorporation of nutrients or bioactive compounds. In parallel with the demand for innovative products incorporating functional ingredients, the utilization of natural antimicrobials meets consumers’ desires for synthetic preservative-free foods with a ‘fresh-like’ quality. Some studies have demonstrated the antimicrobial activity of some polyphenolic compounds or extracts in fruit juices [7,8,9].

Ferulic acid (4-hydroxy-3-methoxycinnamic acid, C_10_H_10_O_4_, FA) is one of the most abundant phenolic acids in plants and acts as a cross-linking agent in cell walls [10]. High concentrations of FA may occur in common foods, such as wheat bran (1358–2293 mg/100 g), corn bran (2610–3300 mg/100 g), and maize bran (3000 mg/100 g), but in general, fruits do not have such amounts, e.g., orange (9.2–9.9 mg/100 g), and apples (0.27–0.85 mg/100 g) [11,12,13]. It has been demonstrated that ferulic acid possesses a broad spectrum of antimicrobial activity, exhibiting effects against yeasts and Gram-positive and Gram-negative bacteria, including *Escherichia coli*, *Klebsiella pneumoniae*, *Enterobacter aerogenes*, *Citrobacter koseri*, *Pseudomonas aeruginosa*, *Helicobacter pylori*, and *Shigella sonnei* [14,15,16]. In addition to its antimicrobial effects, FA has a wide range of biomedical effects, including antioxidant, antimicrobial, antiviral, anti-inflammatory, antiallergic, hepatoprotective, anticarcinogenic, vasodilatory, and antithrombotic effects, and helps increase the viability of sperm [17].

Recently, we demonstrated that the use of FA alone or in combination with aloe vera and alginate coatings inhibited the growth of *L. monocytogenes* on fresh-cut apples [18,19,20] and increased the antioxidant activity (FRAP and DPPH values) and total polyphenol content. The application of a solution of FA prevented browning of fresh-cut taro (*Colocasia esculenta*) and Chinese water chestnut (*Eleocharis tuberosa*) during the cold storage period [21,22]. Therefore, the incorporation of FA in a fruit beverage could serve two purposes: the first is to serve as a vehicle for a functional compound, and the second is to ensure its safety and extend its shelf-life.

The objective of this study was to investigate the antilisterial effects of FA on fresh, ready-to-eat orange and apple juices and evaluate its effects on their microbiological, physicochemical, nutritional, and sensory properties.

## 2. Materials and Methods

### 2.1. Materials

Golden Delicious apples and Navel oranges were obtained from local providers and maintained at 5 ± 2 °C until processing.

Transferulic acid (trans-4-Hydroxy-3-methoxycinnamic acid, ≥99% purity, FA) was obtained from Sigma-Aldrich (ref. W518301, Steinheim, Germany). Tryptone soy broth (TSB), tryptone soy agar (TSA), yeast extract, Palcam base agar, and Palcam selective supplement for *Listeria*, plate count agar (PCA), dichloran rose bengale chloramphenicol agar (DRBC), potassium bisulfate, sodium chloride, and peptone were purchased from Biokar Diagnostics (Allonne, France). Dey–Engley broth was obtained from Honeywell Fluka (Madrid, Spain).

Ascorbic acid, gallic acid, 2,4,6-tris(2-pyridyl)-s-triazine (TPTZ), 2,2-diphenyl-1-picrylhydrazyl (DPPH), and sodium carbonate were purchased from Sigma-Aldrich. Methanol, ethanol, hydrochloric acid (37%), sodium acetate, sodium hydroxide, potassium chloride, ferric chloride hexahydrate, and Folin–Ciocalteu’s reagent were purchased from Panreac (Llinars del Vallès, Spain).

### 2.2. Strains and Inoculum Preparation

A three-strain cocktail of *L. monocytogenes*, serovar 1/2 (CECT 4031), serovar 4b (CECT 935) and serovar 1/2a, which was previously isolated in our laboratory from a fresh-cut lettuce sample [23], was used in this study. Each individual strain was grown for 22–24 h in 50 mL of tryptone yeast broth (TSB; Biokar Diagnostics, Beauvois, France) supplemented with 6 g/L yeast extract (TSBYE) at 37 ± 1 °C in a rotatory shaker set at 150 rpm. Afterwards, 10 mL of each culture were mixed and centrifuged at 9800× *g* at 10 °C for 10 min. The pellet was suspended in saline solution (SS, 8.5 g/L NaCl) to obtain a concentrated suspension, which was approximately 10^10^ cfu/mL. The inoculum concentration was checked by plating appropriate dilutions on Palcam agar and TSAYE. The plates were incubated at 37 ± 1 °C for 48 ± 2 h.

### 2.3. Fresh Apple and Orange Juice Preparation

Cold fruits (5 ± 2 °C) were washed and disinfected with sodium hypochlorite (100 ppm, pH 6.5, 2 min), rinsed in tap water, peeled in an orange peeler (Pelamatic, Valencia, Spain), and cut into pieces. The orange pieces were crushed three times in a pulp and juice extractor (Robot coupe C80 A, Montceau-en-Bourgogne, France). Apple pieces (cv. Golden) were crushed in a cold-press extractor (Infiny Press Revolution, Moulinex, Alençon, France). Each fruit juice sample was split into two batches and maintained in ice water. One batch was left as a control, and 1500 mg/L FA (7.72 mM) were added to the other batch. The FA dose was chosen on the basis of its antimicrobial activity [15,16]. Both batches were blended with a hand blender for 30 s. Fresh orange (FOJ) and apple (FAJ) juices, with or without FA (control, CT), were distributed in sterile tubes and stored at 4 ± 1 °C for subsequent analysis. For each juice before inoculation, a sample was taken for the analysis of pH, titratable acidity, and soluble solids content, as explained below (Section 2.5).

### 2.4. Effect of FA on the Survival of L. monocytogenes in Fresh Orange and Apple Juices

Fresh juices, prepared as described above, were inoculated with the concentrated suspension of *L. monocytogenes* to obtain an initial concentration of approximately 10^4^ cfu/mL. Juice was divided into 15 mL screw-cap tubes and stored at 4 ± 1 °C. Initially, and after 2, 5, 7, and 9 days of storage, the pH and *L. monocytogenes* population were determined by the plating method in triplicate samples (3 tubes). A 10-fold dilution of the juices was performed in saline peptone (SP, 8.5 g/L NaCl, and 1 g/L peptone) and plated in duplicate in Palcam medium for *L. monocytogenes* determination. The plates were incubated at 37 °C for 24–48 h, and typical colonies were counted. The detection limit was 5 cfu/mL. In parallel, 1 mL of the juice was diluted in 9 mL of Dey–Engley medium, followed by incubation at 37 °C to determine the presence of *L. monocytogenes* in the case of counts below the detection limit. The presence of *L. monocytogenes* was confirmed by streaking yellow-colored DE tubes in Palcam medium, followed by incubation at 37 °C.

The results were calculated as cfu/mL of juice, and the data were transformed to log_10_ cfu/mL. After microbial count, the pH of the juices was also determined at each sampling time (pH meter model GLP22, Crison, Alella, Barcelona, Spain), which was equipped with a pH probe (ref. 5203 Hach, Vésenaz, Switzerland).

### 2.5. Effects of FA on the Microbiological, Physicochemical, Nutritional, and Sensory Qualities of Fresh Orange and Apple Juices

Fresh juices were prepared as described above and stored in 50 mL polypropylene conical tubes (Deltalab, Rubí, Spain) at 4 ± 1 °C. Initially, after 2, 6, 8, and 10 days of storage, microbial (total aerobic mesophilic and psychrotrophic microorganisms, molds, and yeasts), physicochemical (total soluble solids, titratable acidity, pH, and color), nutritional (total polyphenol content, antioxidant capacity), and sensory analyses were performed in triplicate. The experiment was repeated two times.

Microbial quality was evaluated by determining the total aerobic mesophilic and psychrotrophic counts (TAM and TAP) and molds, and yeast (M&Y) populations. A 10-fold dilution of the juices was performed in SP, and dilutions were plated in duplicate in Plate Count Agar (PCA, Biokar Diagnostics) for TAM and TAP and Dichloran Rose Bengal Chloramphenicol agar (DRBC, Biokar Diagnostics) for M&Y counts. The plates were incubated at 30 ± 1 °C for 72 h, 6.5 ± 1 °C for 10 days, and at 25 ± 1 °C for 5 days for TAM, TAP, and M&Y counts, respectively.

Total soluble sugar (TSS) was measured at 20 °C with a refractometer (Atago Co. Ltd., Tokyo, Japan), and the results are expressed in °Brix. Titratable acidity (TA) was determined by titration. Ten milliliters of fruit juice were diluted with 10 mL of distilled water and titrated with 0.1 M NaOH until the pH reached 8.2. TA was expressed in g of citric or acid malic/L juice for orange and apple juice, respectively. pH was determined using a pH-meter (model GLP22, Crison, Alella, Barcelona, Spain) equipped with a pH probe (ref. 5203, Hach, Vésenaz, Switzerland). Three measurements were performed in each repetition.

The color of the juices was measured using a CR-200 Minolta Chroma Meter (Minolta, Inc., Tokyo, Japan). Color was expressed in CIE L*, a*, and b* coordinates, using the D65 illuminant and a 10° observer angle. These values were used to calculate the total color difference (ΔE or TCD):

$$\Delta E=\sqrt{{\left(\Delta L^{*}\right)}^{2}+{\left(\Delta a^{*}\right)}^{2}+{\left(\Delta b^{*}\right)}^{2}}$$

where ΔL*, Δa*, and Δb* are the differences between the L*, a*, and b* coordinates in the control and FA juices, respectively. Differences in perceivable color can be analytically classified as very distinct (ΔE* > 3, detected by the human eye), distinct (1.5 < ΔE* < 3, minor color differences can be detected), or small (ΔE* < 1.5), not detected by the human eye [24].

Moreover, a sample was frozen at −80 ± 2 °C for the determination of phenolic acid content and antioxidant activity. The antioxidant capacity was determined by a DPPH· scavenging radical assay (n = 3). The total phenolic content (TPC) was determined using the Folin–Ciocalteu method (n = 3). For the extraction, 3.0 ± 0.1 mL were mixed with 10 mL of methanol 70% (*v*/*v*) and homogenized in a vortex. The samples were immediately placed on a stirrer at 4 °C at 195 rpm for 20 min and centrifuged using a Sigma-3-18 KS centrifuge (Sigma Laborzentrifugen GmbH, Osterode am Harz, Germany) at 13,500× *g* for 20 min at 4 °C. The supernatant was then filtered and brought to 12.5 mL with 70% methanol. DPPH· and TPC determinations were performed as described previously [25]. The antioxidant capacity results were expressed as mg of ascorbic acid equivalents/100 mL (mg AAE/100 mL). The TPC results were expressed as mg of ferulic acid equivalents/100 mL of juice (mg FAE/100 mL juice).

### 2.6. Sensory Analysis

Sensorial evaluation was performed with 30 semi-trained consumers recruited from the IRTA Fruitcentre (Lleida, Spain). They were considered semi-trained because they were all familiar with the quality attributes of juices and could distinguish differences and communicate their reactions, although they were not formally trained. Sensory evaluation was conducted in a sensory laboratory with separate booths. FA-enriched and non-enriched FOJ and FAJ were placed in opaque cups and immediately presented to the consumers on the first day of the experiment. Mineral water was used as a palate cleanser between tastings. All evaluations were conducted in individual booths under ‘red’ lightning at room temperature. Each consumer assessed orange and apple juice samples separately. They were asked to indicate their degree of liking or disliking using a 9-point hedonic scale (1—extremely dislike to 9—extremely like). The experimental procedure was authorized by the Sensorial Sciences and Consumers Committee (CCSC) of the Institute of Agrifood Research and Technology (IRTA).

### 2.7. Statistical Analysis

The microbial population was expressed as log_10_ cfu/mL. All the data were checked for significant differences by applying the General Linear Model (GLM). The criterion for statistical significance was *p* < 0.05. When significant differences were observed, Duncan’s Multiple Range Test (differences among storage times) and *t*-test (differences between treatments) were applied using the SAS system (version 7.4, SAS Institute Inc., Cary, NC, USA).

## 3. Results

### 3.1. Effect of FA in the Survival of L. monocytogenes in Fresh Orange and Apple Juices

The pH of the fresh orange juice was 3.69, the titratable acidity was 7.04 g citric acid/L, and the total soluble solids was 8.63 °Brix, whereas the FA-containing orange juices had values of 3.65, 7.63, and 8.80 °Brix, respectively (Table 1). The initial parameters of apple juice without FA were 3.68, 3.59 g malic acid/L, and 11.3 °Brix and 3.69, 4.11 g malic acid/L, and 11.43 °Brix when FA was added. Therefore, the addition of FA slightly increased the acidity of the juices. The pH of both juices did not significantly change during the storage period.

**Figure 1:**
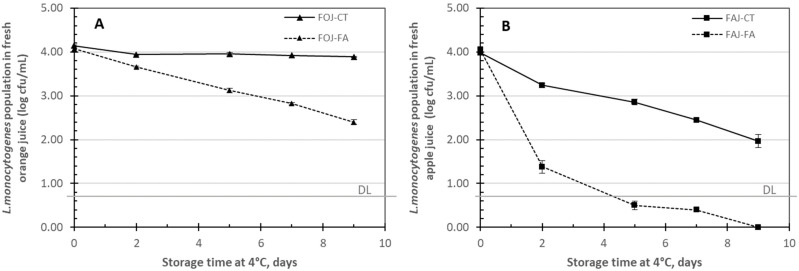
*L. monocytogenes* population in fresh orange juice (**A**) and fresh apple juice (**B**) with (FOJ-FA, FAJ-FA) or without (FOJ-CT, FAJ-CT) ferulic acid at 1500 mg/L and stored at 4 °C for 9 days. The values are the means of three samples. The vertical bars indicate the standard error of the mean; if they are not visible, they are smaller than the symbol size. DL: detection limit (0.70 log cfu/mL).

The initial population of *L. monocytogenes* in FOJ and FAJ was approximately 4 log cfu/mL (Figure 1). In FOJ without FA (FOJ-CT), the *L. monocytogenes* population was maintained during storage at 4 °C, whereas in FOJ containing FA (FOJ-FA), the population progressively decreased to 1.5 log cfu/mL after 9 days of storage (Figure 1A). Regarding FAJ (Figure 1B), the *L. monocytogenes* population decreased by approximately 2.0 log units after 9 days of storage at 4 °C in the control (FAJ-CT). This decrease was more pronounced when FA was added to the juice (FAJ-FA), where the reduction observed was approximately 2.0 log units after 2 days of storage, was below the detection limit after 7 days, and was eliminated after 9 days of storage in the FAJ containing 1500 mg FA/L.

### 3.2. Effects of FA on the Microbiological, Physicochemical, and Nutritional Qualities of Fresh Orange and Apple Juices

#### 3.2.1. Microbiological Quality

**Figure 2:**
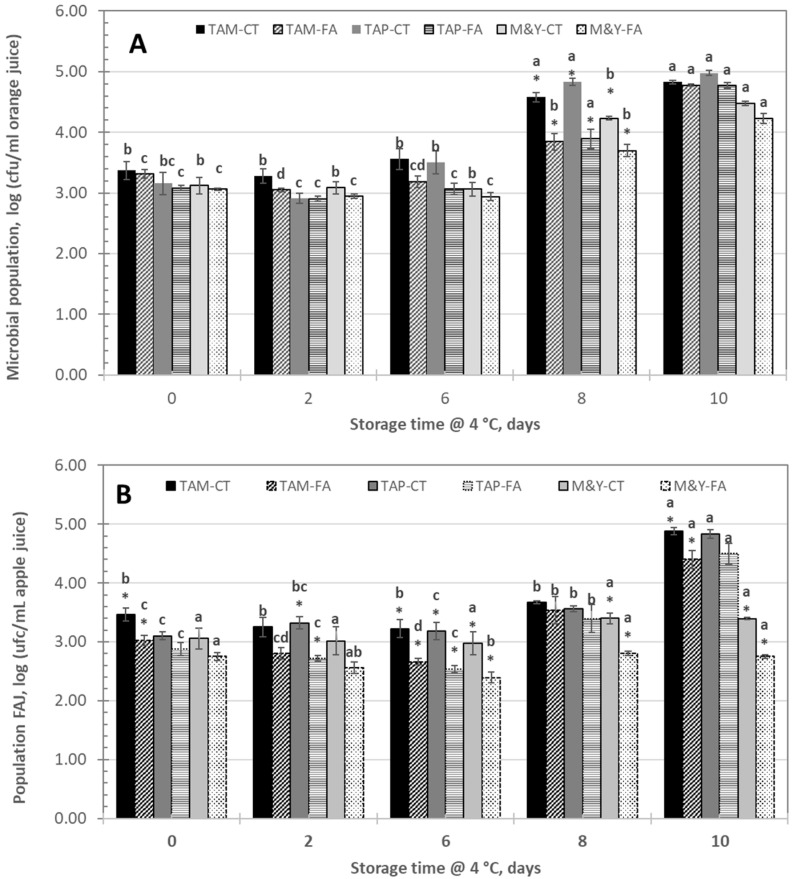
Populations of total aerobic mesophilic (TAM) or psychrotrophic (TPA) microorganisms and molds and yeasts (M&Y) in (**A**) fresh orange juice (FOJ) and (**B**) fresh apple juice without ferulic acid (CT) or with 1500 mg/L ferulic acid (FA) stored at 4 °C. The values are the means of at least three values, and the vertical bars indicate the standard error of the mean. For each storage time and microorganism group, * indicates significant differences due to the presence of FA. For each microorganism group and treatment, different letters indicate significant differences among storage times, according to a Duncan test (*p* < 0.05).

The initial populations of total aerobic mesophilic (TAM), psychrotrophic (TAP), and molds and yeasts (M&Y) in FOJ were 3.4, 3.2, and 3.1 log cfu/mL, respectively (Figure 2A), and were generally maintained during the first 6 days of storage, regardless of the presence of FA. After 8 days of storage, the populations of the studied microorganisms significantly increased, reaching values close to 5.0 log cfu/mL for TAM and TAP and 4.5 for M&Y at the end of the experiment. There were only significant differences in the populations of TAM, TAP, and M&Y between juices containing or not containing FA after 8 days of storage.

In FAJ (Figure 2B), the initial populations of TAM, TAP, and M&Y were 3.5, 3.1, and 3.1 log cfu/mL, respectively, on samples without FA (FAJ-CT). After 6 days of storage, there were significant differences between FAJ-containing FA and the control for all the studied groups of microorganisms. At the end of storage, TAM and TAP increased significantly, reaching values close to 5.0 log cfu/mL. FA prevented the growth of M&Y in FAJ.

#### 3.2.2. Physicochemical Quality

The results revealed that neither the pH nor the TSS content varied with increasing storage time regardless of the addition of FA. The TSS values ranged from 11.9 to 12.2 and between 11.5 and 12.1 °Brix in orange and apple juice, respectively. The pH of the orange juice ranged from 3.96 to 4.17 for FOJ and from 3.92 to 4.07 for FAJ. In contrast, TA was, in general, higher in FA-enriched juices than in CT-juices (Table 2). The apple juice (g malic acid/L) was less acidic than the orange juice (g citric acid/L). The TA of the control juices increased during storage, whereas it was maintained in the FA-enriched juices.

**Figure 3:**
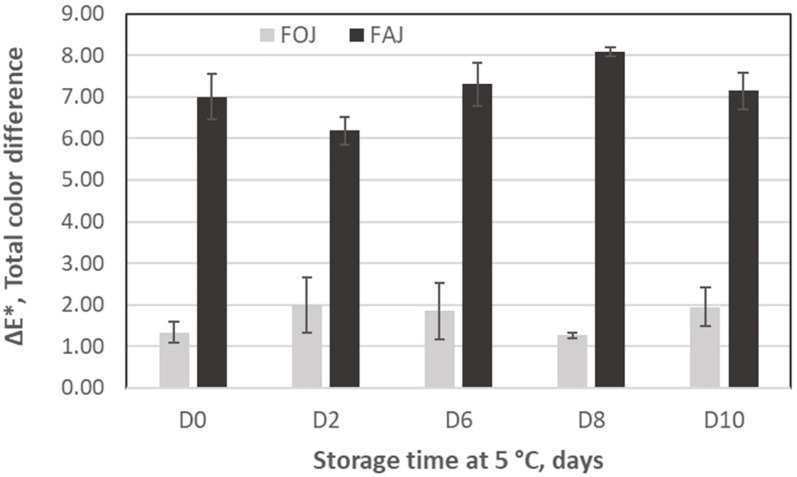
Total color difference (ΔE*) between fresh orange juice (FOJ) and fresh apple juice (FAJ) due to the addition of ferulic acid. The samples were stored at 5 °C for 10 days. The vertical bars indicate the standard deviation of the mean.

**Figure 4:**
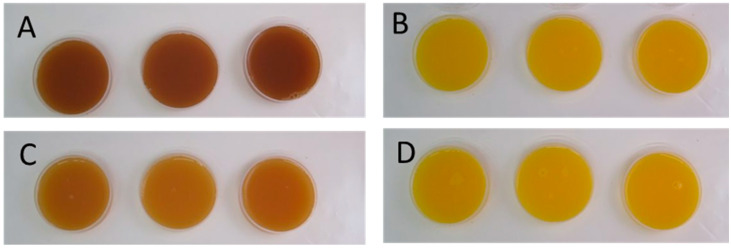
Images of apple (**A**,**C**) and orange (**B**,**D**) juices without FA (**A**,**B**) or with FA (**C**,**D**).

In FOJ, the addition of FA caused significant differences in L*, a*, and b* values throughout storage time (Table S1) in most of the sampling points. However, in relation to total color difference (ΔE*, Figure 3), these differences are considered minor and not perceived by human eyes (ΔE* < 2) and are in accordance with the visual appearance shown in Figure 4. L*, a*, and b* values of FAJ-CT and FAJ-FA were also significantly different throughout the storage period (Table S2). The L* value did not significantly change during storage in FAJ-FA while it increased in the FAJ without ferulic acid. L* a* and b* values in FAJ-CT were significantly different from those in FAJ-FA, and the values indicated greener, redder, and less light (browner) color. The BI was also significantly higher in FAJ-CT than in FAJ containing FA. Moreover, in FAJ, ΔE* values were >6 during the whole storage period, meaning that the color was very distinct and differences were perceived by the human eye (Figure 4). As shown in the images, although FAJ with FA was less brown and brighter than the CT, FA did not completely prevent browning.

#### 3.2.3. Nutritional Quality of Juices

**Figure 5:**
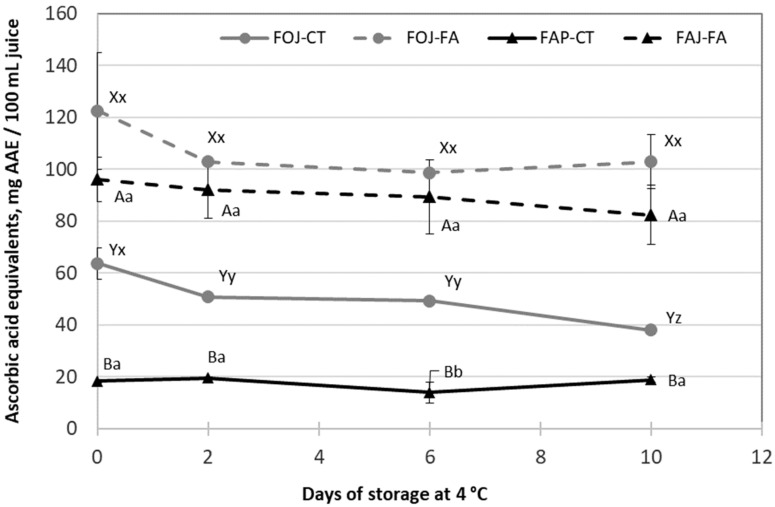
Antioxidant capacity (ascorbic acid equivalents, mg/100 mL juice) measured by the DPPH· method in fresh orange juice (FOJ, gray lines) and fresh apple juice (FAJ, black lines) with (dotted lines) or without (solid lines) the addition of ferulic acid (FA). The samples were stored at 5 °C for 10 days. The vertical bars indicate the standard deviation of the mean, and where not visible, they are smaller than the symbol size. Within each fruit juice and evaluation time, different capital letters indicate significant differences according to a *t*-test. For each treatment, different lower-case letters indicate significant differences according to the storage time.

The antioxidant capacity of FOJ and FAJ significantly increased with the addition of FA (Figure 5), being approximately 2-fold and 5-fold greater, respectively. Moreover, the antioxidant capacity was retained throughout the storage time, except in FOJ without FA (FOJ-CT). Orange juice had a greater antioxidant capacity than apple juice.

**Figure 6:**
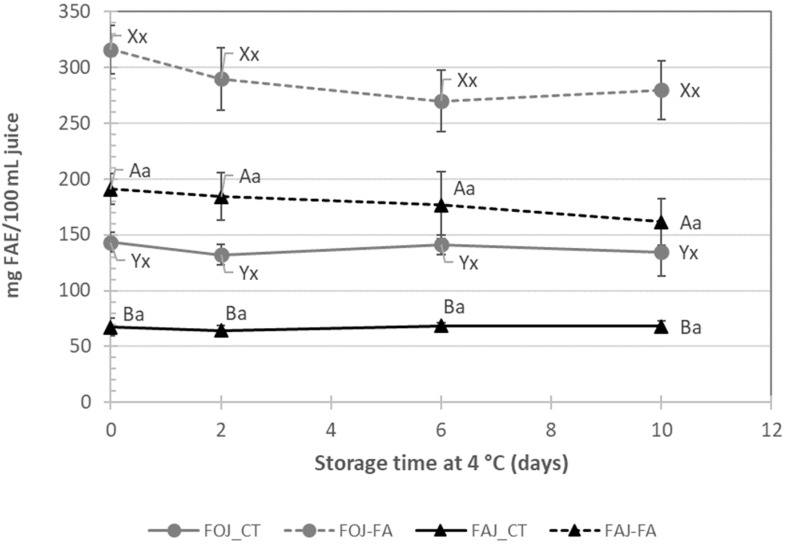
Total phenolic content (ferulic acid equivalents, mg/100 mL juice) of fresh orange juice (FOJ, gray lines) and fresh apple juice (FAJ, black lines) with (dotted lines) or without (solid lines) the addition of ferulic acid (FA). The samples were stored at 5 °C for 10 days. The vertical bars indicate the standard deviation of the mean and, where not visible, they are smaller than the symbol size. Within each fruit juice and evaluation time, different capital letters indicate significant differences according to a *t*-test. For each treatment, different lower-case letters indicate significant differences according to the storage time.

Similar results were obtained for the total phenolic content (TPC, Figure 6), with increases of 2.2 and 2.8 times the EFA in orange and apple juices containing ferulic acid, respectively. Moreover, the TPC was maintained during storage at 4 °C for 10 days. The TPC was greater in orange juice than in apple juice.

#### 3.2.4. Sensory Analysis

**Figure 7:**
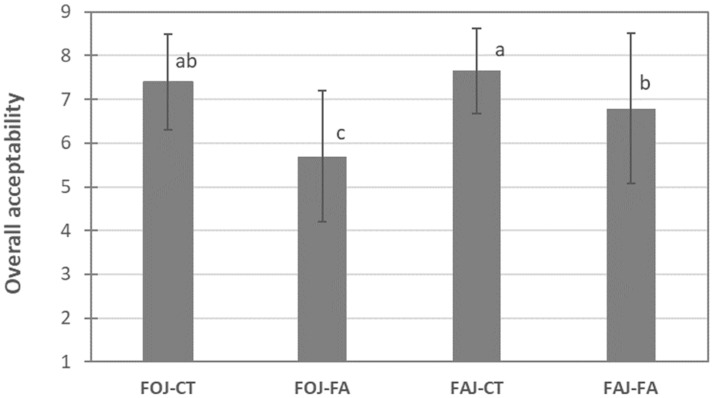
Global acceptability of fresh apple and orange juices with or without the addition of 1500 mg FA/L. Scores represent the mean value of a 1 to 9 hedonic scale. For each fruit, different letters indicate significant differences according to a *t*-test (*p* < 0.05).

Sensory trials demonstrated that fresh juices containing FA were scored lower than the controls were (CT) (Figure 7), and, in general, consumers liked them slightly or moderately (6–7 score). With respect to orange juice, the CT sample had a score of 7.4, with 82.7% of the consumers who liked the juice moderately or very much (score of 7 or higher), whereas the FA-enriched juice had a score of 5.7; only 34.5% of the consumers liked the juice, and 44.8% neither liked nor disliked it (scored 5 or lower). Apple juices were scored higher than orange juices were; 86.2% of consumers scored the FAJ-CT 7 or higher, with a mean of 7.7. The addition of FA significantly decreased its global acceptance, with 58.6% of consumers scoring it equal to or above 7 (mean of 6.8). In general, the acceptance of the FA-enriched juices was above the limit of commercialization (score of 5), but some negative sensations were perceived by some consumers.

## 4. Discussion

In recent decades, the global market demand for healthy beverages has grown tremendously. As consumers become more aware about the health benefits of beverages, researchers and industry have focused on researching functional ingredients to develop new functional beverages. Concomitantly, the use of natural antimicrobials to preserve them is in line with consumer demand for minimally processed food. Within this framework, we aimed to use the antimicrobial and antioxidant properties of ferulic acid to ensure microbial safety and increase the functionality of fresh juices of apples and oranges.

*L. monocytogenes* was the target foodborne pathogen included in this study, as it represents a problem in ready-to-use refrigerated products. It has been demonstrated that even under low pH and refrigeration conditions, *L. monocytogenes* survived in FOJ for 9 days of storage at 4 °C, whereas it decreased in apple juice. This could be explained by the differences in the predominant organic acid (malic vs. citric acid) and polyphenol profiles, as the pH was similar (3.7), and the titratable acidity was greater in orange (7.04 g citric acid/L) juice than in apple juice (3.59 g malic acid/L). FA in both juices increased the reduction in the population of *L. monocytogenes* artificially inoculated, with higher antimicrobial activity in apple juice than in orange juice. FA at 1500 mg/L (7.72 mM) completely eliminated *L. monocytogenes* in apple juice after 9 days of storage. FA has previously been reported to have antimicrobial effects against *L. monocytogenes* in emulsified systems [26] or in DMSO solutions [16]. In BHI broth, the minimum inhibitory concentration of FA against *L. monocytogenes* was found to be pH dependent, with a lower MIC at pH 5 (2.5 mM) than at pH 7 (10 mM) [27]. No effect of 1 mM FA against *L. innocua* or *E. coli* O157:H7 in a model clarified juice was found, but its effect was synergistic when it was combined with UV-A light, resulting in a 5-log reduction in 10 min [28,29]. A synergistic effect of FA combined with UV-A light to reduce foodborne pathogens and spoilage microorganisms in nutrient broth has also been reported [29]. Other phenolic acids, such as vanillic acid at 10 mM, led to a 5-log reduction in *E. coli* O157:H7 in apple juice, but it negatively affected the sensorial acceptance of the juice [7].

In contrast, FA failed to control the growth of indigenous microbiota, as the reduction in mesophiles, psychrophiles, molds, and yeast in FAJ and FOJ containing ferulic acid was lower than 0.6 log units. Previous studies by our group [19] revealed that FA incorporated into Aloe vera coatings at 10,000 mg/L reduced the population of *L. monocytogenes* on fresh-cut apples after 7 days of storage at 5 °C but was also ineffective at reducing the number of *Saccharomyces cerevisiae*. The mode of action of FA involves two mechanisms. First, the intercellular dissociation of the acid causes acidification of the cell cytoplasm and the efflux of K+ ions, leading to the eventual death of the microorganism. Second, the intercalation of the acid into the phospholipid layers of the membrane of the microorganism could also inhibit the transport of substrates used by the key enzymes of the microorganism [26]. Borges et al. [16] demonstrated that FA treatment caused irreversible changes in membrane properties, affecting charge, intra- and extracellular permeability, and physicochemical properties through changes in hydrophobicity, a decrease in negative surface charge, and the occurrence of local rupture or pore formation in the cell membranes, with consequent leakage of essential intracellular constituents in *E. coli*, *P. aeruginosa*, *S. aureus*, and *L. monocytogenes*. Therefore, owing to the different cell wall structures of bacteria, yeasts, and molds, the antimicrobial effects of ferulic acid may differ. Nevertheless, the total aerobic count did not surpass the 6.0 log cfu/mL recommended for juices [30] during storage (10 days).

The results obtained showed that neither the pH nor the TSS content varied with increasing storage time regardless of the addition of FA. Similar results were reported by Nicolau-Lapeña et al. [19,20] for fresh-cut apples and melon. In contrast, TA was, in general, higher in FA-enriched juices than in CT juices. The addition of FA affected the color of the juices, with a total color difference (ΔE*) ranging between 1.271 and 1.949 and between 6.187 and 8.087 for the orange and apple juices, respectively. According to Adekunte et al. [24], perceptual color differences can be analytically classified as very distinct (ΔE* > 3, detectable by the human eye), distinct (1.5 < ΔE* < 3, minor color differences can be detected), or small (ΔE* < 1.5), not detectable by the human eye. Therefore, these differences could be perceived by consumers in apple juice but not in orange juice, as shown in Figure 4. In other vegetable matrices, such as fresh-cut taro (*Colocasia esculenta*) and Chinese water chestnut (*Eleocharis tuberosa*), the application of a solution of 10 mM FA prevented browning during the cold storage period [21,22], probably by suppressing PPO activity, while it did not prevent browning of fresh-cut apples [20].

The fortification of orange and apple juices with FA resulted in a significant increase in the antioxidant potential, leading to DPPH· content in orange and apple juices that were approximately 2- and 5-fold greater than those in raw juices, respectively. This retention was maintained throughout the storage of both fortified products despite the simultaneous short reduction observed on day 2. The TPC, expressed as mg of ferulic acid equivalents/100 mL in fresh, non-fortified juices, was greater in the orange juice than in the apple juice, as previously reported by Mattila et al. [12]. The TPC values in fortified orange and apple juices were approximately 2- and 4-fold greater, respectively, than those in fresh products on day 0 of storage, since FA is a phenolic compound with high antioxidant activity. The clear correlation between the DPPH· and TPC values in this study indicated that the addition of FA to fresh liquid juices led to products with high antioxidant capacity after 10 days of refrigerated storage. Fortification with FA could represent a potential strategy not only to have a bacteriostatic effect on added products but also to promote health benefits because of its reported anti-inflammatory, antithrombosis, and anticancer activities [17,31]. This fortification strategy has been explored in other fruit products, such as fresh-cut apples and melons, which deliver products with increased antioxidant activity [19,20]. Similarly, due to its antimicrobial activity along with its potent antioxidant and radical scavenging properties, FA has enormous applicability in the food and cosmetic industries [32] and is already used in several countries as an active ingredient in many skin lotions, sunscreens, and pharmaceutical preparations [33]. However, the consumers’ overall acceptance of both juices decreased with the addition of FA, with final scores above the limit of acceptability (>5), which could be related to the unpleasant bitter or astringent flavor of this phenolic acid [34] and the increase in total acidity.

Further studies could be carried out to evaluate the encapsulation of FA to diminish unpleasant flavors perceived by consumers and favor solubility, as in previous works such as gallic acid within zein ultrafine fibers [35] and vanillic acid into layered double hydroxide [36]. In juices, different plant extracts have been encapsulated for stabilization [37]. Our work explored the feasibility of using FA not only for its antimicrobial effects but also for increasing its nutritional composition, particularly its antioxidant and polyphenol content. The addition of FA significantly increased the antioxidant activity and total phenolic content of the fresh juices. In contrast, the TPC of freshly cut taro soaked in a 10 mM solution of ferulic acid was lower than that of the control. Notably, the application of FA to fresh-cut fruits or vegetables by immersion completely differs from the addition of FA to juice studied in our work.

## 5. Conclusions

In summary, our findings suggest that the application of FA could be an effective approach for maintaining the safety of both fresh apple and orange juices. This treatment exhibited antilisterial activity while preserving color without adversely impacting sensory attributes. Additionally, we observed a significant increase in antioxidant activity and phenolic content, suggesting potential nutritional advantages. Further studies should be carried out to explore in depth its potential for industrial application, including the use of ferulic acid extracts from the cereal industry.

## Figures and Tables

**Table 1:** Quality parameters of fresh apple and orange juices with or without ferulic acid (FA, 1500 mg/L) in the inoculation experiments.

Juice	Treatment	pH	Acidity (g Acid Citric or Malic/L)	SS (°Brix)
Orange	Control	3.69	7.04	8.63
	FA	3.65	7.63	8.80
Apple	Control	3.68	3.59	11.3
	FA	3.69	4.11	11.4

**Table 2:** Titratable acidity (TA, g citric acid/L orange juice and g malic acid/L apple juice) of fresh orange juice (FOJ) and fresh apple juice (FAJ) enriched (FA) or not enriched (CT) with ferulic acid (FA) during storage at 4 °C. The values are the means of three replications. For each juice and column, different letters indicate significant differences among storage times (*p* < 0.05). For each juice, * indicates a significant difference (*p* < 0.05) due to FA enrichment according to a *t*-test. For each column, different letters indicate significant differences among storage days according to a Duncan test (*p* < 0.05).

	Day	TA (g/L)	
		CT	FA
**FOJ**	0	c 4.92 *	a 5.98 *
	2	ab 5.03 *	a 5.93 *
	6	ab 5.17 *	a 5.84 *
	8	bc 5.05 *	a 5.79 *
	10	a 5.28 *	a 6.04 *
**FAJ**	0	c 2.15	a 2.59
	2	c 2.19 *	a 2.98 *
	6	b 2.30 *	a 2.85 *
	8	b 2.30 *	a 2.89 *
	10	a 2.39 *	a 2.89 *

## Data Availability

The original contributions presented in the study are included in the article/Supplementary Materials, further inquiries can be directed to the corresponding author.

## Supplementary Materials

The following supporting information can be downloaded at: https://www.mdpi.com/article/10.3390/foods13203288/s1, Table S1: Cielab parameters (L*, a*, b*) and browning index (BI = [100*(x − 0.31)]/0.172, where x = (a* + 1.75L*)/(5.645L* + a* − 0.3012b*)) of FOJ without FA (FOJ-CT) or with 1500 mg/L of FA (FOJ-FA) after processing (0) and after storage at 4 °C; Table S2: Cielab parameters (L*, a*, b*) and browning index (BI = [100*(x − 0.31)]/0.172, where x = (a* + 1.75L*)/(5.645L* + a* − 0.3012b*)) of FAJ without FA (FAJ-CT) or with 1500 mg/L of FA (FAJ-FA) after processing (0) and after storage at 4 °C.
