# Supplementary material for: Fortification of Orange and Apple Juices with Ferulic Acid: Implications for Food Safety and Quality
Source: Foods. 2024 Oct 16;13(20):3288. doi: 10.3390/foods13203288 (PMC11507299; doi:10.3390/foods13203288)
Supplement: Supplementary file 1 [file foods-13-03288-s001.zip › foods-3225277-supplementary.pdf]

Table S1. Cielab parameters (L*, a*, b*) and browning index (BI = [100 * (x – 0.31)]/0.172, where x = (a*+1.75L*)/(5.645L* + a* - 0.3012 b*)) of FOJ without FA (FOJ-CT) or with 1500 mg/L of FA (FOJ-FA) after processing (0) and after storage at 4 °C. For each treatment and color parameter, different letters indicate significant differences among storage time. For each parameter and storage time, an * indicates significant differences in relation to the addition of FA.

		Storage time at 4 °C, days				
	Treatment	0	2	6	8	10
L*	CT	44.075c*	44.553b*	44.140c*	44.910a*	44.780ab*
	FA	45.393b*	46.023a*	46.151a*	45.971a*	45.982a*
a*	CT	2.494b	2.714a*	1.878c*	2.503b	2.609ab*
	FA	2.617a	2.539a*	2.356b*	2.522a	2.299b*
b*	CT	23.115b*	23.191b*	22.527c*	24.362a	24.088a
	FA	23.700c*	24.702a*	23.844bc*	24.622a	24.247ab
BI	CT	75.463b	75.078b*	71.693c*	78.844a	78.162a*
	FA	75.112bc	77.694a*	73.563c*	77.471a	75.474b*

Table S2. Cielab parameters (L*, a*, b*) and browning index (BI = [100 * (x – 0.31)]/0.172, where x = (a*+1.75L*)/(5.645L* + a* - 0.3012 b*)) of FAJ without FA (FAJ-CT) or with 1500 mg/L of FA (FAJ-FA) after processing (0) and after storage at 4 °C. For each treatment and color parameter, different letters indicate significant differences among storage time. For each parameter and storage time, an * indicates significant differences in relation to the addition of FA.

		Storage time at 4 °C, days				
	Treatment	0	2	6	8	10
L*	CT	32.097c*	33.131b*	32.960b*	33.073b*	33.349a*
	FA	38.634*	38.592*	38.982*	39.356*	39.382*
a*	CT	6.633a*	6.164bc*	6.413ab*	6.300bc*	6.048c*
	FA	4.716a*	4.645ab*	4.353bc*	4.319bc*	4.044c*
b*	CT	7.985b*	8.150ab*	8.056b*	8.336a*	8.331a*
	FA	10.316b*	10.338b*	10.900ab*	11.126a*	11.500a*
BI	CT	43.598a*	41.393b*	41.791b	42.511ab*	41.557b
	FA	39.540b*	39.531b*	40.481ab	40.769ab*	41.513a
